# Standardized Mortality Ratio and Long-Term Stroke Incidence After PFO Closure: A Register Study

**DOI:** 10.1016/j.jacadv.2025.102469

**Published:** 2025-12-19

**Authors:** Maria Angerbjörn, Bengt Johansson, Elisabeth Hahlin, Daniel Rinnström, Camilla Sandberg, Christina Christersson, Peder Sörensson, Mikael Dellborg, Ulf Thilén, Signild Åsberg, Johanna Pennlert

**Affiliations:** a Department of Public Health and Clinical Medicine, Umeå University, Umeå, Sweden; b Department of Diagnostics and Intervention Sciences, Umeå University, Umeå, Sweden; c Department of Community Medicine and Rehabilitation, Umeå University, Umeå, Sweden; d Department of Medical Sciences, Cardiology, Uppsala University, Uppsala, Sweden; e Department of Medicine Solna, Karolinska Institute Stockholm, Solna, Sweden; f Department of Molecular and Clinical Medicine, Institute of Medicine, Sahlgrenska Academy, University of Gothenburg, Gothenburg, Sweden; g Department of Cardiology, Clinical Sciences, Skane University Hospital, Malmö, Sweden; h Department of Medical Sciences, Uppsala University, Uppsala, Sweden

**Keywords:** cryptogenic stroke, device closure, patent foramen ovale, PFO closure, SMR, standardized mortality ratio

## Abstract

**Background:**

Closure of a patent foramen ovale (PFO) is frequently recommended in patients with cryptogenic stroke. Long-term outcomes in real-world settings remain unknown.

**Objectives:**

This study analyzed standardized mortality ratio (SMR), subsequent stroke, and associated risk factors after PFO closure.

**Methods:**

National registers on congenital heart disease and stroke were cross-linked to identify individuals who underwent PFO closure between 2001 and 2018. The ratio of observed to expected deaths was calculated (SMR). Data were analyzed using survival analysis and Cox regression.

**Results:**

A total of 827 patients (60.5% males, median age 47.9 years, IQR: 40.0-55.6 at the time of PFO closure) were included and observed for a median duration of 8.0 years (IQR: 4.8-11.0). During follow-up, 23 patients died, SMR was 0.65 (95% CI: 0.41-0.98). A total of 34 ischemic strokes occurred, yielding an incidence rate of 0.51 events per 100 patient-years. Among the 34 patients who experienced a subsequent stroke, 27 were receiving antithrombotic therapy at the time of the event. New-onset atrial fibrillation following PFO closure was associated with an increased risk of subsequent ischemic stroke (HR: 8.2; 95% CI: 2.6-25.8), as was active/previous smoking (HR: 2.5; 95% CI: 1.2-5.1).

**Conclusions:**

Patients undergoing PFO closure demonstrated a lower all-cause mortality compared to the general population. The observed rate of subsequent ischemic stroke was consistent with findings from previous randomized controlled trials. New-onset atrial fibrillation following PFO closure and active/previous smoking emerged as modifiable risk factors.

In cryptogenic stroke, a patent foramen ovale (PFO) may be present and represents a potential route for paradoxical embolism. Four randomized clinical trials have demonstrated that PFO closure reduces the risk of subsequent stroke events compared to medical therapy in patients with cryptogenic stroke, using various occluder devices.^1^, ^2^, ^3^, ^4^ These positive trials were preceded by earlier studies that failed to show a significant benefit of PFO closure over medical treatment.^5^, ^6^, ^7^ Improved statistical power and stricter inclusion criteria were key factors distinguishing the positive trials from the negative ones.

The prevalence of recurrent stroke has varied across the clinical trials demonstrating the benefit of PFO closure.^1^, ^2^, ^3^, ^4^ Based on the cumulative evidence from these randomized controlled trials, current guidelines recommend PFO closure in patients with cryptogenic stroke and a PFO.^8^, ^9^, ^10^ However, substantial heterogeneity exists among the trials with respect to study design, choice of occluder devices, and antithrombotic treatment protocols. The duration of follow-up in these trials is relatively limited, raising questions regarding the long-term efficacy of PFO closure. Moreover, a considerable proportion of patients were lost to follow-up in the positive clinical trials.^1^, ^2^, ^3^, ^4^ The implementation of trial-based therapies into real-world clinical practice may yield differing results regarding efficacy.^11^, ^12^, ^13^, ^14^, ^15^, ^16^, ^17^, ^18^, ^19^ A key question remains whether the incidence of subsequent stroke events following PFO closure in a real-world setting aligns with the favorable outcomes observed in clinical trials.

Several observational studies of PFO closure in routine clinical practice have reported a high prevalence of cardiovascular risk factors—such as arterial hypertension, hypercholesterolemia, and smoking—among treated patients at baseline.^15^^,^^16^^,^^18^ New-onset atrial fibrillation (AF) has also been frequently observed, though its impact on the risk of subsequent stroke following PFO closure remains uncertain.^15^, ^16^, ^17^, ^18^^,^^20^, ^21^, ^22^ AF is the most commonly reported adverse event associated with PFO closure and is generally considered to be transient.^23^ However, its true prevalence, and whether it may have been present before device implantation, remains difficult to determine due to the often paroxysmal nature of the condition.

Following ischemic stroke or transitory ischemic attack (TIA), the identification and management of cardiovascular risk factors is a central component of secondary prevention, and has been demonstrated to reduce the risk of recurrence in clinical trials.^24^ Most patients with cryptogenic stroke, including those undergoing PFO closure, receive some form of antithrombotic therapy. However, postclosure antithrombotic management is not well studied, and clinical practice varies widely. Guideline recommendations are based on limited evidence.^9^^,^^25^ A short initial period of dual antiplatelet therapy is generally advised, but longer-term strategies are not well defined.^9^^,^^25^

Data from observational studies comparing PFO closure with medical therapy suggest a lower mortality rate among patients undergoing closure.^11^ Whether the long-term survival of patients treated with PFO closure differs from that of the general population remains unknown. In this register-based analysis, we utilized national health care and mortality registers to assess all-cause mortality, subsequent ischemic stroke events following PFO closure, and factors associated with subsequent stroke among patients with a history of PFO closure.

## Methods

### The SWEDCON register (SWEDish register on CONgenital heart disease)

The nationwide SWEDCON register aims to follow individuals with congenital heart disease from childhood into adulthood.^26^ Established in 1992, it has since 1998 included data from all 7 health care regions in Sweden. As of 2017, SWEDCON contained information on approximately 14,000 adults (defined as age ≥18 years) with congenital heart disease. All PFO closure procedures performed in adults with a history of cryptogenic stroke or TIA are recorded in SWEDCON. Data collection is conducted at each participating center and includes detailed information on diagnoses, interventions, electrocardiography (ECG), echocardiography, medications, and other clinical variables.^27^ Mortality data are updated monthly based on official statistics.^26^

### The Swedish stroke register

The Swedish stroke register (Riksstroke) was established in 1994 and has provided nationwide coverage of all Swedish hospitals admitting patients with acute stroke since 1998.^28^ A validation study published in 2016 estimated the completeness of the register to exceed 90%, with interhospital reliability ≥85%.^29^ Riksstroke is the world's longest-running national stroke register and includes data on the acute phase, acute treatment, cardiovascular risk factors, secondary prevention, and follow-up.^28^ Patients aged ≥18 years who are hospitalized with a diagnosis of ischemic stroke, intracerebral hemorrhage, or unspecified stroke are eligible for inclusion. Since its inception, the register has documented data on more than 490,000 patients (Riksstroke Annual Report 2017, available at www.riksstroke.se).

### Study population

The study population consisted of patients ≥18 years of age who had undergone endovascular PFO closure, as recorded in the SWEDCON register, prior to October 5, 2017, when data were extracted. Index stroke was identified in register data for 531 of the 827 patients, all of which were classified as ischemic strokes. The remaining 296 patients had no registered index stroke; some of them might be transient ischemic attacks. To identify PFO closure procedures and subsequent ischemic stroke events, SWEDCON data were cross-linked with Riksstroke, capturing outcomes up to December 31, 2018. TIA as an outcome was not included.

Patients who had undergone PFO closure before January 1, 2001—prior to nationwide coverage and quality assurance in both registers—were excluded to ensure the reliability of follow-up data. Diagnoses, echocardiographic findings, and procedural records in SWEDCON were reviewed to confirm both the diagnosis of PFO and the indication for closure. Patients lacking either a confirmed PFO diagnosis or documentation of PFO closure were excluded. The study was approved by the Swedish Ethical Review Board (Etikprövningsnämnden), reference numbers: Dnr 08-218M, 2017-431-32M, and 2019-01053.

### Definition of variables

Baseline characteristics were obtained from the initial clinical visit to a PFO closure center or from Riksstroke at the time of admission for the index stroke. AF at baseline was defined as either a prior diagnosis of AF or flutter, or as atrial arrhythmia observed on an ECG. Arterial hypertension was defined as a clinical blood pressure ≥140/90 mm Hg, a documented diagnosis of hypertension, or ongoing treatment with antihypertensive medications. Left ventricular ejection fraction >50% was considered normal. Body mass index (BMI) was dichotomized as >25 or ≤25 kg/m^2^. Lipid-lowering therapy was defined based on a recorded register entry indicating active treatment at baseline. New-onset AF was identified from ECG findings documented during follow-up visits at participating clinical centers following PFO closure.

### Standardized mortality ratio

The standardized mortality ratio (SMR) was calculated as the ratio of observed deaths in the study cohort to the number of expected deaths in the general population. Expected mortality was estimated using age-, sex-, calendar year-, and country-specific mortality rates derived from the Human Mortality Database. These rates were multiplied by the total person-time at risk. An SMR >1 indicates a higher mortality rate compared to the general population, and vice versa. Ninety-five percent CIs were calculated using the Garwood method, which is based on the relationship between the Poisson distribution and the chi-square distribution.^30^ An SMR was considered statistically significant when the 95% CI did not include 1.

### Statistical analyses

Missing data were imputed using multiple imputation in SPSS with 50 imputations. Kaplan-Meier survival curves were used to illustrate stroke-free survival and to estimate the cumulative incidence of the primary endpoint: ischemic stroke following PFO closure. A secondary composite endpoint comprising subsequent stroke or death was also evaluated. Cox proportional hazards regression was used to identify associations between baseline variables and clinical outcomes. Variables deemed clinically relevant and satisfying the proportional hazards assumption were included in univariable Cox regression analyses. Age and sex were included as strata in the model. Death was considered a competing event of stroke, and we modeled the cause-specific hazard of stroke by censoring observations at the time of death.

Statistical significance was defined as a 2-sided *P* value <0.05. All analyses were conducted using IBM SPSS Statistics for Windows, version 28.0.1.1 (IBM Corp). The study adhered to the STROBE (Strengthening the Reporting of Observational Studies in Epidemiology) guidelines for cohort studies.^31^

## Results

The study cohort consisted of 827 patients (60.5% male) who had undergone percutaneous closure of a PFO following cryptogenic stroke or TIA. The median age at the time of closure was 47.9 years (IQR: 40.0-55.6). The total follow-up time amounted to 6,633 person-years, with a median follow-up duration of 8.0 years (IQR: 4.8-11.0). Baseline comorbidities included AF (0.4%), diabetes mellitus (1.5%), arterial hypertension (34.1%), BMI >25 kg/m^2^ (45.0%), and active/previous smoking (26.4%) (Table 1).

**Table 1:** Baseline Characteristics of the Study Population

	Total (N = 827)	Ischemic Stroke After PFO Closure (n = 34)	Missing Data, n
Median age at PFO closure (IQR)	47.9 (40.0-55.6)	49.5 (40.9-58.1)	0
Age <40 y, n (%)	205 (24.8)	7 (20.6)	0
Age 40-50 y, n (%)	271 (32.8)	10 (29.4)	0
Age 50-60 y, n (%)	235 (28.4)	11 (32.4)	0
Age >60 y, n (%)	116 (14.0)	6 (17.6)	0
Women, n (%)	327 (39.5)	14 (41.2)	0
Men, n (%)	500 (60.5)	20 (58.8)	0
Atrial fibrillation before closure, n (%)	3 (0.4)	0 (0)	0
Atrial fibrillation, new-onset after closure, n (%)	18 (2.2)	4 (11.8)	0
Hypertension, n (%)	282 (34.1)	13 (38.2)	20 (2.4)
Active or previous smoking, n (%)	218 (26.4)	15 (44.1)	0
Diabetes, n (%)	12 (1.5)	1 (2.9)	0
BMI >25 kg/m^2^, n (%)	372 (45.0)	20 (58.8)	114 (13.8)
Lipid-lowering treatment, n (%)	280 (33.9)	12 (35.3)	21 (2.5)
Type of device (%)			50 (6.0)
Amplatzer PFO, n (%)	299 (36.2)	13 (38.2)	
Amplatzer ASD, n (%)	89 (10.8)	6 (17.6)	
Helex, n (%)	182 (22.0)	5 (14.7)	
Solysafe, n (%)	4 (0.5)	0 (0)	
Other, n (%)	203 (24.5)	8 (23.5)	
Device size >25 mm (%)	247 (29.9)		66 (8.0)
Device size, stratified (%)			66 (8.0)
<10 mm, n (%)	6 (0.7)	0 (0)	
10-20 mm, n (%)	145 (17.5)	8 (23.5)	
21-30 mm, n (%)	474 (57.3)	16 (47.1)	
>31 mm, n (%)	136 (16.4)	7 (20.6)	
Procedural time			87 (10.5)
≤100 min, n (%)	695 (84.0)	27 (79.4)	
>100 min, n (%)	45 (5.4)	4 (11.8)	

PFO = patent foramen ovale; BMI = body mass index; ASD = atrial septal defect.

A total of 23 patients died during follow-up, 3 of whom had experienced a subsequent ischemic stroke prior to death. The overall mortality rate was 0.35 deaths per 100 person-years. Case fatality, defined as death within 28 days following a stroke, was 0%. One patient died 2 months after a subsequent ischemic stroke. The SMR, comparing observed mortality in the study population with that of the general Swedish population, was 0.65 (95% CI: 0.41-0.98).

During follow-up, 34 patients experienced a subsequent ischemic stroke following PFO closure, corresponding to an incidence rate of 0.51 events per 100 person-years. The median time to subsequent stroke was 3.1 years (range: 0.01-13.5). Three patients experienced a stroke within 28 days after PFO closure, and these were classified as procedure-related complications (0.36% of all PFO closures). The median age at subsequent stroke was 53.9 years (IQR: 45.4-65.6). At the time of subsequent stroke, 27 out of 34 patients were receiving antithrombotic treatment: 22 were on antiplatelet therapy, 3 on anticoagulation, and 2 on both.

The cumulative incidence of subsequent ischemic stroke was 0.5% at 1 year, 1.1% at 2 years, 2.9% at 5 years, and 4.6% at 10 years. No statistically significant difference in the incidence of subsequent stroke was observed between sexes (log-rank *P* = 0.8). The cumulative incidence of the composite outcome—subsequent ischemic stroke or death—was 0.6% at 1 year, 1.5% at 2 years, 4.1% at 5 years, and 7.5% at 10 years, again with no sex differences (log-rank *P* = 0.8).

**Figure 1:**
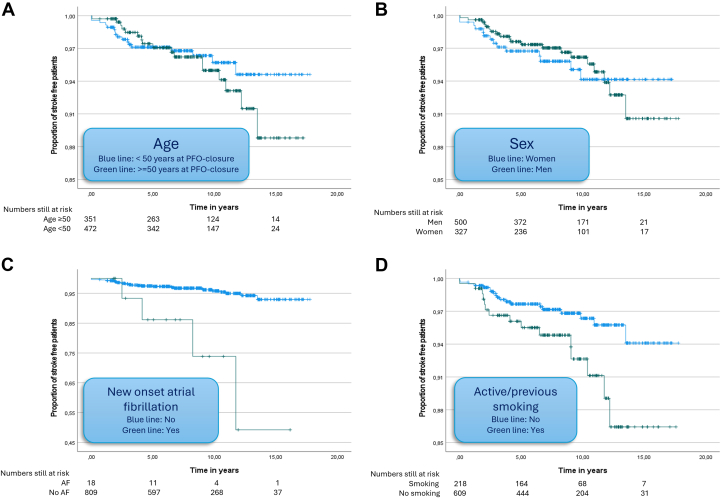
**Freedom From Stroke After PFO Closure** Kaplan-Meier curves of time from PFO closure to subsequent ischemic stroke stratified by (A) age, (B) sex, (C) new-onset atrial fibrillation, and (D) active/previous smoking. AF = atrial fibrillation; PFO = patent foramen ovale.

When stratified by age, stroke-free survival declined more rapidly among patients younger than 50 years at baseline compared to those aged 50 years and older during the first 6 years of follow-up; thereafter, the survival curves crossed, and the decline became steeper in the older age group. A similar pattern was observed between sexes: women exhibited a steeper decline in stroke-free survival during the first 12 years of follow-up, after which the survival curve crossed, and men experienced a faster decline (Figure 1). For the composite endpoint of stroke or death, older patients (≥50 years) had a consistently steeper decline over time, while no notable sex differences were observed (see Supplemental Material).

In univariable Cox regression analysis, new-onset AF after PFO closure (HR: 7.9; 95% CI: 2.7-23.0) and active/previous smoking (HR: 2.3; 95% CI: 1.2-4.5) were associated with increased risk of subsequent stroke. In multivariable analysis, both variables remained independently associated with subsequent stroke: new-onset AF (HR: 8.2; 95% CI: 2.6-25.8) and active/previous smoking (HR: 2.5; 95% CI: 1.2-5.1) (Table 2).

**Table 2:** Hazards of Subsequent Ischemic Stroke After PFO Closure in Presence of Different Covariates

	Hazards of Subsequent Ischemic Stroke After PFO Closure			
	Unadjusted Hazards		Adjusted Hazards Stratified by Age and Sex	
	HR (95% CI)	*P* Value	HR (95% CI)	*P* Value
New-onset atrial fibrillation	7.9 (2.7-23.0)	<0.001	8.2 (2.6-25.8)	<0.001
Active/previous smoking	2.3 (1.2-4.5)	0.018	2.5 (1.2-5.1)	0.010
BMI >25 kg/m^2^	2.1 (0.9-4.6)	0.078	2.1 (0.9-4.9)	0.074
Hypertension	1.1 (0.5-2.3)	0.855	0.9 (0.4-1.9)	0.722
Diabetes	2.5 (0.3-18.6)	0.366	2.2 (0.3-18.7)	0.456
Lipid-lowering treatment	1.4 (0.7-2.9)	0.362	1.2 (0.6-2.7)	0.574
Type of device				
Amplatzer PFO	Ref.			
Amplatzer ASD	2.0 (0.7-5.5)	0.165	1.5 (0.5-4.4)	0.499
Helex	0.8 (0.3-2.2)	0.645	0.8 (0.3-2.4)	0.701
Solysafe	Low frequency		Low frequency	
Other	1.5 (0.6-3.8)	0.349	1.5 (0.6-4.0)	0.379
Size of device >25 mm	1.1 (0.5-2.3)	0.815	1.1 (0.5-2.5)	0.796
Procedural time >100 min	2.0 (0.7-6.1)	0.216	1.8 (0.6-5.6)	0.301

Univariable and multivariable Cox regression analyses of associations between covariates and subsequent ischemic stroke, including stratification for sex and age under and over 50 years. All covariate counts reflect baseline (= PFO closure) except “new-onset atrial fibrillation.”

Abbreviations as in Table 1.

New-onset AF occurred in 18 patients (2.2%) following PFO closure, with a median time to diagnosis of 19.5 days (range: 0-174 days). Notably, 82% of cases were diagnosed within 45 days of the procedure. During follow-up, 4 patients with new-onset AF experienced a subsequent ischemic stroke. Closure devices in these patients included the Amplatzer PFO Occluder (n = 1), Helex Septal Occluder (n = 1), and other or unspecified devices (n = 2). Stroke events in these individuals occurred between 2 and 12 years after closure and were thus not classified as procedural complications (Central Illustration).

**Central Illustration:**
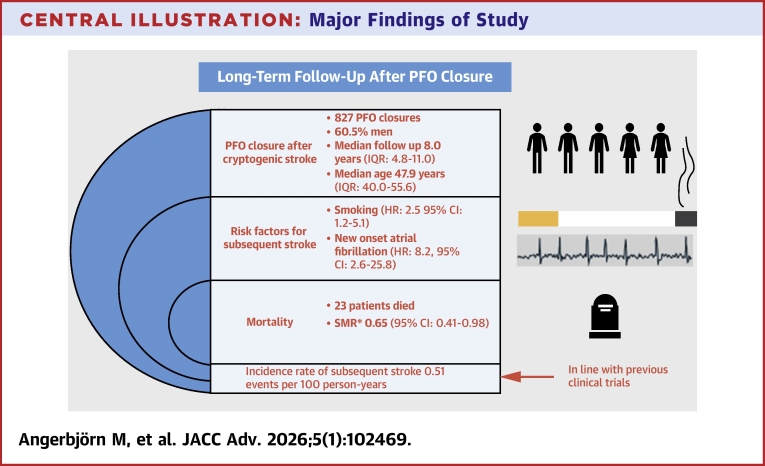
Major Findings of Study SMR = standardized mortality ratio.

Device type was documented in 69.5% of the total cohort, reported as “other” in 24.5%, and missing in 6.0%. The Amplatzer PFO Occluder was the most commonly used device (36.2%), and most devices (57.3%) were within the 21 to 30 mm size range. In 84.0% of cases, procedural time was <100 minutes. Procedural outcomes were documented in 776 patients; of these, 757 (97.6%) had a good procedural result, 8 (1.0%) had a partially good result, and the procedure was discontinued in 11 patients (1.4%). Among the discontinued procedures, 10 were successfully repeated at a later date. Of the 34 patients who experienced a subsequent stroke, one belonged to the “partially good” group, one to the “discontinued” group, 2 had missing procedural data, and the remaining 30 (88.2%) were among those with an initial “good” procedural result.

## Discussion

In this nationwide cohort study, we found that the SMR was lower in patients with cryptogenic stroke or TIA who underwent PFO closure compared to the general population. This observation suggests that the pathophysiological mechanisms underlying cryptogenic stroke differ from those of stroke with known etiology, where traditional cardiovascular risk factors are more prominent. Nonetheless, smoking remained independently associated with subsequent stroke, and BMI showed a borderline association, indicating that conventional risk factors still play a role in long-term outcomes following PFO closure.

We hypothesize that the lower mortality rate observed may reflect a generally better cardiovascular risk profile among patients selected for PFO closure, along with more intensive secondary prevention measures. Furthermore, the incidence of subsequent ischemic stroke in this real-world setting was consistent with rates reported in randomized clinical trials, supporting the effectiveness of PFO closure when performed according to trial-based criteria. Importantly, our data underscore the relevance of guideline-concordant patient selection and follow-up, as subsequent stroke despite closure was not negligible, and AF was implicated in nearly 12% of subsequent stroke events.

Although death could theoretically preclude the detection of subsequent strokes, we observed that stroke occurred more frequently than death, and Kaplan-Meier curves for the composite outcome of stroke or death did not indicate significant underestimation of subsequent strokes due to mortality. The overall incidence rate of subsequent ischemic stroke (0.51 events per 100 patient-years) closely aligns with that reported in pivotal trials (eg, 0.58 events per 100 patient-years),^2^ and our cumulative incidence of 1.1% at 2 years lies within the range reported by major studies (0-1.4%).^1^^,^^3^^,^^4^ These findings suggest that during the study period (2001-2018), clinical practice in Sweden, although conservative and predating the publication of pivotal trials, yielded rates of subsequent strokes following PFO closure comparable to those seen in controlled trial settings.

Compared to other observational studies, our study offers several advantages. The use of the Riksstroke ensures near-complete case ascertainment, minimizing the limitations often associated with administrative data or self-reported outcomes. Moreover, our cohort encompasses the vast majority of PFO closures performed nationwide during the study period, enhancing the generalizability of the results. To our knowledge, this is the first study to report the SMR in a PFO closure population, with our finding of an SMR of 0.65 suggesting that patients undergoing closure may benefit a survival advantage—possibly due to a combination of patient selection, improved cardiovascular management, and structured post-stroke follow-up.

New-onset AF occurred in 2.2% of patients, with the majority of cases detected within 45 days of the procedure. Four of these patients later experienced an ischemic stroke, and AF was associated with subsequent stroke in both univariable and multivariable analyses. Although clinical trials have consistently reported higher AF incidence in the PFO closure arms,^1^^,^^3^^,^^6^ only one study demonstrated a link to stroke recurrence^20^—a finding that may have been device-specific, as that trial used a device later withdrawn. Our longer follow-up (median 8.0 years) provides novel insights and raises several important questions: Does the procedure itself induce more AF than previously thought? Is the risk for AF following PFO closure device-dependent? Could subsequent strokes following PFO closure be further reduced by preventing or more aggressively managing postclosure AF? Alternatively, might periprocedural AF simply unmask a predisposition in a susceptible subset of patients? Further studies are needed to clarify these mechanisms. Until then, our findings support close clinical monitoring in cases where new-onset AF is detected postclosure.

A relevant point of comparison is a recent Swedish observational study using administrative data to compare PFO closure with medical therapy,^32^ likely involving a partially overlapping population. While both studies report similar mortality and rates of subsequent strokes following PFO closure, key differences lie in the granularity of data. The present study includes only PFOs, whereas the comparator study included all atrial septal communications, including atrial septal defects (ASDs), which are generally larger and associated with greater hemodynamic load. We hypothesize that increased stroke risk in the ASD group may stem not only from paradoxical embolism but also from arrhythmias or impaired atrial function secondary to volume overload or surgical scarring. Our exclusion of ASDs and focus on precisely classified PFOs enhances the internal validity of the present results.

Clinical trials have generally included patients with high likelihood of PFO-related stroke, as reflected by a mean RoPE (Risk of Paradoxical embolism) score of ∼7.3.^1^ In real-world settings, lower RoPE scores (6.0-7.0) have been reported,^15^^,^^16^^,^^19^ reflecting a trend toward broader patient inclusion. While direct RoPE scoring was not possible in our cohort due to data limitations, we estimate an average score of 6.8 based on literature benchmarks—suggesting our cohort is representative of real-world practice, yet still comparable to trial populations. These findings underscore the importance of maintaining strict adherence to evidence-based indications to optimize outcomes.

Our survival analyses revealed a crossover effect in incidence of subsequent stroke by age and sex. Initially, patients younger than 50 years and women had a steeper decline in stroke-free survival, but beyond 6 to 12 years, this pattern reversed. These observations may reflect age- and sex-specific differences in stroke etiology: hormonal factors may contribute more to early events in younger women, while cumulative cardiovascular risk may drive later stroke events in older individuals, particularly men. This crossover effect violated the proportional hazards assumption, preventing the calculation of meaningful HRs for age and sex. While prior studies have reported conflicting associations between age and subsequent strokes, none have found a significant relationship with sex.^16^, ^17^, ^18^, ^19^ Our findings support a multifactorial model of the events of subsequent stroke following PFO closure, where early and late events may be driven by distinct pathophysiological processes.

In conclusion, this study demonstrates that PFO closure in a real-world setting is associated with low mortality and a rate of subsequent strokes comparable to that reported in clinical trials. Traditional cardiovascular risk factors, especially smoking and AF, remain important predictors of subsequent strokes. The findings highlight the need for careful patient selection, vigilant follow-up for atrial arrhythmias, and adherence to guideline-based indications to optimize outcomes. The observed lower SMR suggests a favorable prognosis in this selected population and merits further investigation.

### Study Limitations

This study is based on data from national health care registers and is therefore limited to the variables available within these sources. Although data coverage and validity are generally high, certain stroke events may be underreported. Specifically, the Riksstroke includes only inpatient cases, thereby excluding severe strokes resulting in prehospital death, strokes in palliative settings, or silent infarctions that do not lead to medical contact. As a result, the incidence of ischemic stroke may be underestimated. TIA was excluded as an outcome due to inherent challenges in its definition and inconsistent reporting over time in the Riksstroke. This exclusion may have led to an underestimation of neurological events during follow-up.

Residual confounding cannot be entirely excluded, as some clinical and imaging-based variables were unavailable or incomplete. While most register data have been validated, the use of unvalidated or proxy variables in some analyses must be acknowledged as a limitation. For example, we lacked sufficient data to accurately calculate individual RoPE or PFO-Associated Stroke Causal Likelihood scores, limiting our ability to stratify stroke recurrence risk based on established prediction models.

Furthermore, detection of postprocedural AF was primarily based on routine ECGs obtained during clinical follow-up. This approach likely underestimates the true prevalence of new-onset AF, as episodes of paroxysmal or asymptomatic AF may go undetected in the absence of continuous or extended rhythm monitoring.

Lastly, the majority of the study period (2001-2018) preceded the widespread clinical implementation of PFO closure following the publication of pivotal trials. As such, the procedure was used restrictively and in carefully selected patients, which may have influenced the observed outcomes and limits the generalizability of results to broader populations treated in more recent years. Temporal changes in stroke care, secondary prevention strategies, and device technology during the study period could not be accounted for within the available data and study design.

## Conclusions

In this nationwide cohort study, we observed a low incidence of subsequent ischemic stroke following PFO closure, consistent with rates reported in clinical trials. These findings suggest that the real-world application of PFO closure in Sweden between 2001 and 2018 achieved outcomes comparable to those observed under trial conditions, despite being performed during a period of more selective use.

The observed SMR of 0.65 supports the notion that patients undergoing PFO closure represent a relatively healthy subgroup with low baseline cardiovascular risk and limited long-term impact from their index stroke or TIA. This favorable mortality outcome highlights the importance of maintaining rigorous patient selection criteria and comprehensive secondary prevention strategies.

New-onset AF occurred in 2.2% of patients and was independently associated with subsequent stroke, underscoring the need for systematic rhythm monitoring and clinical follow-up in this population. Until further evidence clarifies the mechanisms linking PFO closure and AF, careful screening and management of arrhythmias remain essential components of postprocedural care.

Perspectives **COMPETENCY IN MEDICAL KNOWLEDGE:** The novel observation of an SMR of 0.65 in the PFO closure population compared with the general population may have important implications for clinical management and warrants confirmation in future studies. This finding further emphasizes the need to establish and maintain the safety profile of the PFO closure procedure. The observed association between new-onset AF and subsequent stroke identifies a potential target for improvement; however, additional research is required to understand the underlying pathophysiological mechanisms. The identification of smoking as a modifiable risk factor suggests that conventional vascular risk factors contribute to recurrent stroke after PFO closure. Whether this reflects suboptimal candidate selection for closure or the emergence of a new stroke mechanism following a true PFO-related event could not be determined within the scope of this study. The observed postclosure stroke rates, which are consistent with those reported in previous clinical trials, are reassuring. Nevertheless, studies focusing on the period following the 2018 paradigm shift in treatment recommendations are needed to fully evaluate the clinical benefits of PFO closure in contemporary practice.

**TRANSLATIONAL OUTLOOK 1:** The observed findings on AF after PFO closure underscore the need for heightened attention to AF, potentially through more thorough preprocedural evaluation and intensified postprocedural follow-up.

**TRANSLATIONAL OUTLOOK 2:** The observed stroke rates after PFO closure in this patient population are reassuring. Strict adherence to established closure criteria remains essential, and further studies examining the post-2018 period are warranted to confirm that contemporary practice continues to achieve favorable outcomes.

## Funding support and author disclosures

This study was supported by grants from the 10.13039/501100003793Swedish Heart-Lung Foundation, 10.13039/501100004885Umeå University, Region Västerbotten, Strokeforskning Norrland, the Heart Foundation of Northern Sweden, and Visare Norr. The authors have reported that they have no relationships relevant to the contents of this paper to disclose.
