# Supplementary material for: Standardized Mortality Ratio and Long-Term Stroke Incidence After PFO Closure: A Register Study
Source: JACC Adv. 2025 Dec 19;5(1):102469. doi: 10.1016/j.jacadv.2025.102469 (PMC12775957; doi:10.1016/j.jacadv.2025.102469)

**Supplementary material**. Kaplan Meier curves of time from PFO closure to a combined endpoint of subsequent ischemic stroke or death stratified by a, age, b, sex, c, new-onset atrial fibrillation and d, active/previous smoking.

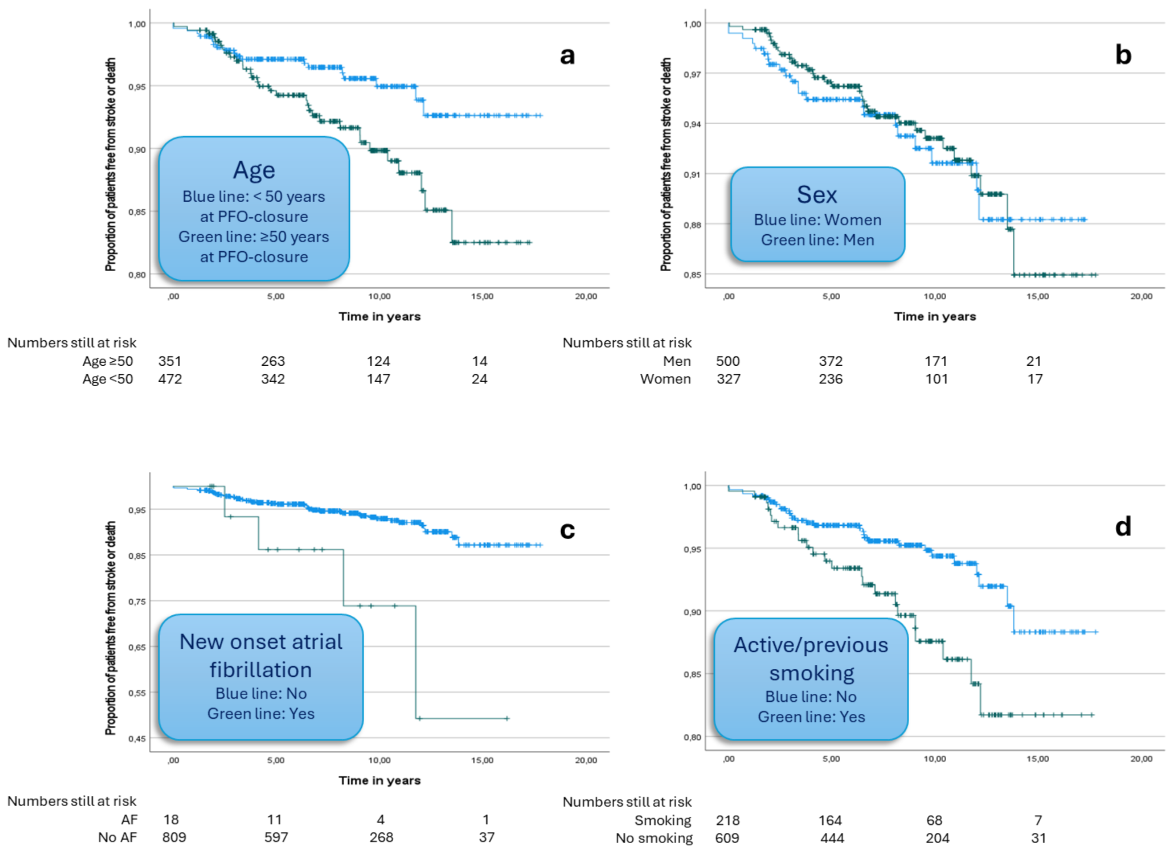

Supplement: Supplementary data [file mmc1.pdf]
